# Early Warning Scores in Patients with Suspected COVID-19 Infection in Emergency Departments

**DOI:** 10.3390/jpm11030170

**Published:** 2021-03-02

**Authors:** Francisco Martín-Rodríguez, José L. Martín-Conty, Ancor Sanz-García, Virginia Carbajosa Rodríguez, Guillermo Ortega Rabbione, Irene Cebrían Ruíz, José R. Oliva Ramos, Enrique Castro Portillo, Begoña Polonio-López, Rodrigo Enríquez de Salamanca Gambarra, Marta Gómez-Escolar Pérez, Raúl López-Izquierdo

**Affiliations:** 1 Advanced Clinical Simulation Centre, Advanced Life Support Unit, Emergency Medical Services, Faculty of Medicine, Universidad de Valladolid, 47005 Valladolid, Spain; fmartin@saludcastillayleon.es; 2 Faculty of Health Sciences, Universidad de Castilla la Mancha, 45600 Talavera de la Reina, Spain; JoseLuis.MartinConty@uclm.es (J.L.M.-C.); Begona.polonio@uclm.es (B.P.-L.); 3 Data Analysis Unit, Health Research Institute, Hospital de la Princesa, Madrid (IIS-IP), C/Diego de León, 62, 28006 Madrid, Spain; agetro.ortega@gmail.com; 4 Emergency Department, Hospital Universitario Rio Hortega de Valladolid, Gerencia Regional de Salud de Castilla y León (SACYL), c/Dulzaina, 2, 47012 Valladolid, Spain; vcarbajosar@saludcastillayleon.es (V.C.R.); icebrianru@saludcastillayleon.es (I.C.R.); jolivara@saludcastillayleon.es (J.R.O.R.); ecastrop@saludcastillayleon.es (E.C.P.); renriquezd@saludcastillayleon.es (R.E.d.S.G.); rlopeziz@saludcastillayleon.es (R.L.-I.); 5 Emergency Medical Services-SACYL, Paseo Hospital Militar, nº 24, 47007 Valladolid, Spain; mgomezescolar@saludcastillayleon.es

**Keywords:** COVID-19, pandemic, early warning score, clinical decision-making, triage, emergency medicine

## Abstract

Early warning scores (EWSs) help prevent and recognize and thereby act as the first signs of clinical and physiological deterioration. The objective of this study is to evaluate different EWSs (National Early Warning Score 2 (NEWS2), quick sequential organ failure assessment score (qSOFA), Modified Rapid Emergency Medicine Score (MREMS) and Rapid Acute Physiology Score (RAPS)) to predict mortality within the first 48 h in patients suspected to have Coronavirus disease 2019 (COVID-19). We conducted a retrospective observational study in patients over 18 years of age who were treated by the advanced life support units and transferred to the emergency departments between March and July of 2020. Each patient was followed for two days registering their final diagnosis and mortality data. A total of 663 patients were included in our study. Early mortality within the first 48 h affected 53 patients (8.3%). The scale with the best capacity to predict early mortality was the National Early Warning Score 2 (NEWS2), with an area under the curve of 0.825 (95% CI: 0.75–0.89). The severe acute respiratory syndrome coronavirus 2 (SARS-CoV-2) positive patients presented an area under the curve (AUC) of 0.804 (95% CI: 0.71–0.89), and the negative ones with an AUC of 0.863 (95% CI: 0.76–0.95). Among the EWSs, NEWS2 presented the best predictive power, even when it was separately applied to patients who tested positive and negative for SARS-CoV-2.

## 1. Introduction

The Coronavirus disease 2019 (COVID-19), an infectious outbreak caused by the severe acute respiratory syndrome coronavirus 2 (SARS-CoV-2), has revolutionized healthcare systems around the world [1,2]. No country was entirely or even partially prepared to face the major consequences of the coronavirus infection, a highly contagious disease with a rapid rate of hospitalization. COVID-19 presents several unique characteristics relating to its symptomatology, treatment, diagnosis, risk factors, medical protocols, etc., which make this pandemic a true challenge to healthcare personnel in their daily work [3]. Then, there is also the mandatory use of personal protective equipment to perform their usual functions under new conditions for which they were not fully prepared [4,5].

Certainly, the current pandemic is a stress test for the healthcare system. The number of patients with suspected COVID-19 infection has overloaded the capacity of healthcare facilities, sometimes confronting health professionals with ethical issues beyond their own duties. Under this complex scenario, requiring a constant adaptation of diagnoses and management as the pandemic progresses, the use of reliable tools to identify the patients who are the most at risk of clinical deterioration is mandatory [6].

The use of diagnostic and/or prognostic scores is a reality in multiple clinical contexts. However, the selected score for specific situations requires the assessment of particular characteristics. Notably, scoring systems such as the sequential organ failure assessment score (SOFA) [7], or specific biomarkers [8], are not intended to be applied under situations with high flows of patients. Instead, for those situations in which a short period of time between patients’ evaluations is required, it is necessary to implement simple, effective and, if possible, noninvasive and continuous scores in order to be able to carry out patient screenings in a timely manner [9].

The early warning scores (EWSs) have high clinical consistency and have been used repeatedly in emergency departments (ED) [10,11]. These scores are based on the weightings of vital and clinical signs that are routinely determined in any patient such as respiratory rate, oxygen saturation, blood pressure, heart rate, temperature, use of supplemental oxygen, level of consciousness and/or age [12].

EWSs are tools that assist in the clinical decision-making process and are implemented in Intensive Care Units (ICUs), non-ICU settings, ED and more recently in pre-hospital care or even nursing homes [13,14,15]. The advantage of this type of score is the possibility of early detection of the risk of clinical deterioration, which has facilitated its use in multiple health systems, with a global implementation [16,17,18]. In addition, EWSs provide alert triggers that are adaptable to virtually any disease, making them highly versatile tools, capable of being used in different environments, physio-pathological situations, healthcare worker trainings or even latitudes [19,20,21,22].

For the COVID-19 pandemic, we hypothesized that EWSs can be an effective tool that will allow an initial triage of patients to be evacuated with high-priority by ambulances to EDs, helping in such complex decision-making situations [23,24,25].

The objective of this study is to determine the prognostic accuracy of four EWSs in high-priority patients taken to EDs in ambulances with suspected COVID-19 infections for predicting risk of deterioration two days after the event; in particular, we assessed the implication of EWSs in confirmed COVID-19 cases.

## 2. Materials and Methods

### 2.1. Study Design, Population and Setting

We conducted a retrospective cohort study between 12 March and 31 July 2020. The study was conducted in the Valladolid Oeste Health Area, integrated by the Rio Hortega Hospital (tertiary university hospital) and seventeen ambulatory health centers with an overall reference population of 269,221 inhabitants, and involving the local Emergency Medical Services (EMSs) with twenty-one ambulances (4 advanced and 17 basic life support), all of which belonged to the Public Health System of Castilla-León (Spain).

### 2.2. Participants

Adult patients with suspected COVID-19 infections who were transferred with high-priority by ambulances to EDs were included in the study. Either a family, nursing home or emergency physician confirmed the suspicion of COVID-19 infection. During the initial evaluation, when it was decided to transfer the patient by ambulance to the ED, the healthcare workers based their evaluations on, among other things, epidemiological criteria and on the search for the following signs and/or symptoms: dyspnea, low oxygen saturation, pathological auscultation, cough, tachypnea, asthenia, arthralgias, costal pain, fever, headache, diarrhea, vomiting, syncope and altered level of consciousness. Patients with cardiorespiratory arrest upon arrival at the ED who arrived by other means of transport (e.g., private ambulance, walking), and cases where it was impossible to calculate the scores due to the absence of all clinical parameters were excluded.

### 2.3. Outcome

The outcome was mortality from any cause within 48 h after the ambulance transfer. This time window was chosen because it is the usual outcome used in this type of study [10,26,27], and because the EWSs used in this study are designed to predict short-term clinical deterioration.

Death data were obtained from a review of the patients’ electronic medical records.

### 2.4. Selection of Early Warning Scales

Among all the EWSs available, we chose those that were the easiest and quickest to use and that could be performed by personnel with minimal training, based on standard measurements.

For this study, we selected four EWSs: (1) the quick sequential organ failure assessment score (qSOFA) [28,29]—to be used for patients with suspected infection outside the intensive care units which can help to detect the sepsis. (2) National Early Warning Score 2 (NEWS2) [27,30]—a score developed to predict the risk of major adverse cardiovascular events, which is validated and widely implemented. (3) Modified Rapid Emergency Medicine Score (MREMS) [31]—this score is similar to NEWS2 but incorporates age as an additional parameter [4]. Rapid Acute Physiology Score (RAPS) [32]—an additional element evaluates the prognostic value of standard parameters together with the mean arterial pressure (see Table 1 with all scoring systems analyzed).

In general terms, each EWS has a warning trigger that indicates if the risk of clinical deterioration is high above this value—e.g., a qSOFA score equal or higher than two points is highly indicative of sepsis, which helps healthcare workers to suspect the presence of sepsis and to continue requesting complementary tests to refine the diagnosis [29,33]. Other scores such as NEWS establish several cutoff points, stratifying the risk in the following levels: (1) low (0–4 points), (2) low-intermediate (3 points in one of the parameters, which indicates maximum weighting of this vital constant), (3) medium (5–6 points) and (4) high (7 or more points, indicating the need for critical support teams) [34,35].

### 2.5. Predictors and Data Abstraction

Covariates included information extracted from the standardized clinical history used by EMS professionals, such as age, sex, type of ambulance (basic or advanced life support), and patients coming from nursing homes. The clinical data necessary to calculate the scores (respiratory rate, oxygen saturation, systolic blood pressure, heart rate, temperature, level of consciousness, use of supplemental oxygen, and mean arterial pressure) were obtained by an emergency registered nurse in the triage box of the ED during the first contact with the patient. Oxygen saturation, blood pressure, temperature, and heart rate were measured using the Connex^®^ Vital Signs Monitor (Welch Allyn, Inc., Skaneateles Falls, NY, USA).

By reviewing the electronic medical record, the following outcomes were obtained: patients with a confirmatory analytical diagnosis of SARS-CoV-2 (polymerase chain reaction test positive), inpatients, mortality data within two days, and finally, comorbidities to calculate the Charlson Age Comorbidity Index (CACI).

Once all the parameters were entered into the database, the scores were calculated using the XLSTAT^®^ BioMED for Microsoft Excel^®^ version 14.4.0 software (Microsoft Inc., Redmond, WA, USA).

### 2.6. Data Analyses

A database of patients was created specifically for this work. Case registrations were further tested to eliminate ambiguous elements and to validate the data collection instrument. Missing values were replaced using the mode of the variable; none of the registered variables presented more than 5% of missing values.

Normality tests were performed on all the quantitative variables which were described as median and interquartile range (25th–75th percentile). Categorical variables were described by using absolute frequencies and percentages.

For the comparison of means of quantitative variables, the Mann–Whitney U-test was used; the Chi-square test was used on 2 × 2 contingency tables of qualitative variables to assess their association or dependency relationship. The Fisher’s exact test was used when it was necessary.

The predictive validities of the EWSs were evaluated by the area under the curve (AUC) of the receiver operating characteristic (ROC). The p value of the hypothesis test (H0: ABC = 0.5) and the AUC 95% confidence interval (CI) were also assessed. Further statistical characteristics such as: positive predictive value, negative predictive value, positive likelihood ratio, negative likelihood ratio, and odds ratios were determined. To determine the cutoff point, the Younden test was used. The resulting AUC of each EWS was compared the other EWS’ AUCs by means of the Delong test.

Additionally, the discrimination capacities of the EWSs were assessed considering the final diagnostic of SARS-CoV-2 (polymerase chain reaction test positive)—that is, the AUC of the ROC was determined for each category of this variable.

All statistical analyses were performed using our own codes and base functions in R, version 4.0.2 (http://www.R-project.org; the R Foundation for Statistical Computing, Vienna, Austria, accessed on 30 September 2020).

## 3. Results

### 3.1. Patient Characteristics

**Figure 1:**
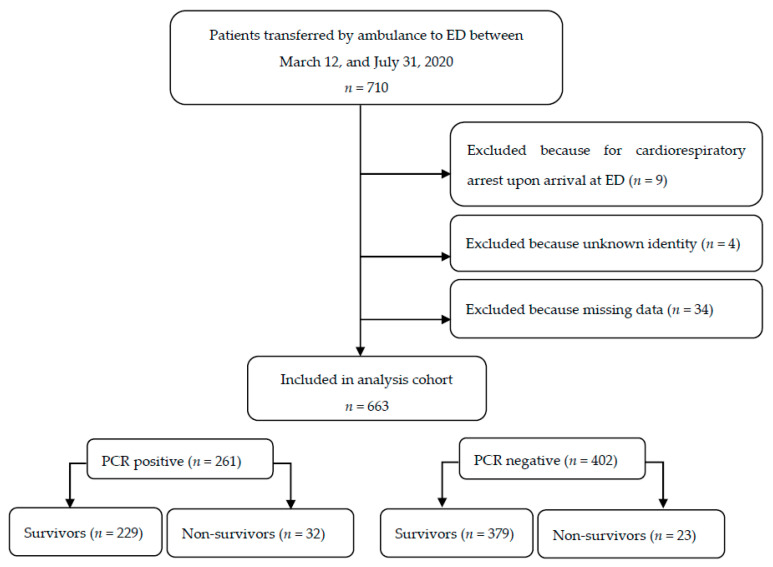
Flowchart showing analysis population. ED: emergency department; PCR: polymerase chain reaction test. Mortality rates refer to total mortality (60-day follow-up).

The total cohort included 663 participants who were recruited based on 21 ambulance stations and transported to an ED (see Figure 1). The median age was 82 years (IQR, 70–88 years); a total of 341 (51.4%) were females. The rate of SARS-CoV-2 confirmed by analytical tests was 39.4% (261 cases). The mortality rate (from any cause) was 8.3% (53 cases) within two days, with a specific mortality due to SARS-CoV-2 of 12.3% (32 cases) (Table 2). A special subcohort was composed of patients coming from nursing homes which accounted for 45.2% (300 cases) of the total; among them, 45.6% SARS-CoV-2 were diagnosed (119 cases), with a two-day mortality of 17.9% (Table 2).

In analyzing the two-day mortality in both confirmed SARS-CoV-2 and non-SARS-CoV-2 cases, it was observed that the respiratory rate, oxygen saturation, supplemental oxygen and Glasgow coma scale presented statistically significant differences (see Table 3). In both cohorts, the median age was significantly higher in nonsurvivors than in the survivors, with a large percentage of deaths of patients from nursing homes.

### 3.2. EWS Discrimination for the Global Cohort

Of all the scores evaluated, the scores of nonsurvivors were significantly higher than those of the survivors (*p* < 0.001 for all cases) (Table 3).

All the EWSs presented good prognostic validities for mortality within 2 days, as observed in Table 4. The best prognostic power was for NEWS2 with an AUC of 0.825 (95% CI: 0.75–0.89; *p* < 0.001). The comparison between AUCs showed that NEWS2 presented statistically significant differences between RAPS and qSOFA but not with the MREMS score (see Table 5).

The corresponding Odds ratio (OR) for each EWS also helped to determine their validity. As mentioned above, all of the scores presented statistically significant p values, with the RAPS score presenting the highest odds ratio (Table 6).

### 3.3. EWS Discrimination for the Global Cohort

The issue of whether being tested positive for SARS-CoV-2 could modify the predictive validities of the EWSs was also addressed. The predictive power was slightly worse for patients who tested positive for SARS-CoV-2, as can be observed in Table 4; the AUC was always lower for SARS-CoV-2 positive patients. For the global cohort, NEWS2 was the best ranked EWS, with an AUC of 0.804 (95% CI: 0.71–0.89; *p* < 0.001) for positive cases and an AUC of 0.863 (95% CI: 0.76–0.95; *p* < 0.001) for patients who tested negative for SARS-CoV-2. In this sense, only NEWS2 vs. qSOFA presented statistically significant differences regarding the AUC for both SARS-CoV-2 positive and negative cases (Table 6). Regarding the OR, there was a higher OR, in all EWSs, for the SARS-CoV-2 negative cohort than the positive cohort (Table 5).

## 4. Discussion

To our knowledge, this is the first retrospective study on a cohort of patients referred with high-priority in ambulances to EDs with suspected COVID-19 infection that analyzes the prognostic precision of different EWSs in detecting the risk of early clinical deterioration with result of two-day mortality.

Of all the scores analyzed, the best overall prognostic performance was obtained by NEWS2. The comparison between SARS-CoV-2 positive and negative patients showed that the scores are slightly more accurate for non-SARS-CoV-2 patients, regardless of age, sex or comorbidities. In this sense, patients with SARS-CoV-2 had a lower saturation and more than double two-day mortality than the non-SARS-CoV-2 patients. Of the scores analyzed, the predictive ability of NEWS2 suggests that it is appropriate for initial short-term prognostic evaluation in patients with suspected COVID-19 infection.

There is growing interest in developing strategies for a fast characterization of patients with SARS-CoV-2. Several studies have analyzed the capacity of different EWSs, with standardized use in EDs to discriminate the short-term prognoses of these patients to decide the best strategy to manage resources as efficiently as possible. Myrstad et al. [36] evaluated the ability of NEWS2 to predict seven-day mortality and Jang et al. [23,37] evaluated the ability of NEWSs and qSOFA to predict 28-day mortality. Covino et al. [23] and Gidari et al. [14] studied whether NEWSs and other scores could predict the need for ICUs or Sixt et al. [38] and Hu et al. [39,40] analyzed the usefulness of NEWS2 at day 7 in relation to hospitalization. All of the aforementioned studies were developed using small cohorts (from 66 to 334 cases), different outcomes and with a limited number of EWSs studied. Our study, on the other hand, analyzed a single and large cohort on which several EWSs were tested by using a single outcome.

However, from using different weights for each variable, all the scores basically evaluated the same set of physiological parameters [41]. Our results suggest that in the group of SARS-CoV-2 positive patients, mortality was associated with respiratory rate, oxygen saturation, the use of supplemental oxygen and a low level of consciousness, whereas in the group of SARS-CoV-2 negative patients, mortality was associated with oxygen saturation, the use of supplemental oxygen, systolic blood pressure, mean blood pressure and level of consciousness. These results indicate that the parameters evaluating ventilatory function would have a greater weight in patients with SARS-CoV-2, whereas in non-SARS-CoV-2 patients, hemodynamic variables would play an important role [25,26]. In this sense, several studies have shown that at the beginning of infection, the hemodynamic status is not particularly altered [42,43], a fact that penalizes scores in this type of patient; in fact, our results showed that all EWSs are better for non-SARS-CoV-2 patients than SARS-CoV-2 positive patients, the latter of which has a lower ability to predict the two-day mortality. Therefore, the little weight that hemodynamic variables have in NEWS2, the lack of initial alteration of hemodynamic status, and the importance that NEWS2 gives to discharges in ventilatory function, make this EWS the one with the best predictive capacity compared to the other EWSs.

The use of EWSs is a common practice in multiple clinical contexts, providing very valuable information that helps characterize more precisely the clinical risk of deterioration and therefore facilitates risk stratification [12,34,44]. In our results, the high-risk cutoff points show that, globally, for the NEWS2 scale, 7 points is where the best joint sensitivity and specificity is obtained—a score that reached 8 points for the SARS-CoV-2 positive population. This fact is in accordance with results from other studies carried out on different populations and health settings [27,45,46].

Healthcare in the current COVID-19 pandemic must necessarily be based on the best use of available resources [24,47]. The number of patients, their different presentations and their condition over time make scoring systems a tool that should certainly be considered [48,49].

This study has several limitations. First, the selection of participants was made by criteria of opportunity among all adult patients transferred with high priority by ambulance to the ED of a tertiary hospital. In order to minimize bias, patients referred by basic and advanced life support from the entire hospital health area were included in the study, from the beginning of the pandemic until the end of July, without distinction between rural or urban area, time of day and day of the week.

Second, our endpoint was two-day mortality, which did not include deceased patients after this time window. Future studies will consider the evolution in the medium-long-term period.

Third, the data extractors were not blinded. To avoid the outcomes being subject to interpretation, the main researcher, once all the data had been collected, reviewed all the cases with a final outcome of death within the first two days in order to minimize the risk of errors.

Fourth, the selection of the analyzed EWSs has necessarily been partial. We are aware of the multitude of scores that can be analyzed, but those described above have been chosen considering timeliness, bibliographic consistency and level of implementation; however, for future studies it is necessary to evaluate the behaviors of other types of scores, such as the Quick COVID-19 severity index (qCSI), a tool designed especially for patients with COVID-19 [50,51].

Finally, the sample size is sufficient for a preliminary study, but it is necessary to promote multicenter prospective studies and in different clinical contexts (e.g., nursing homes, ambulance, ED, ICU) to know, in a broader context, the utility and prognostic precision of the EWSs analyzed.

## 5. Conclusions

In summary, health systems, in the face of the current COVID-19 pandemic, must implement scoring systems that will allow one to discriminate the presence of high-risk patients in a fast, noninvasive and effective way.

Of all the EWSs analyzed, NEWS2 has the best predictive capacity and the highest sensitivity for cases of SARS-CoV-2. According to our results, a patient with SARS-CoV-2 and a NEWS2 score equal to or greater than 8 points presents a high risk of clinical deterioration and a very high risk of two-day mortality.

## Figures and Tables

**Table 1:** Analyzed scores.

**NEWS2**	**3**	**2**	**1**	**0**	**1**	**2**	**3**
Pulse (bpm)	≤40		41–50	51–90	91–110	111–130	≥131
BR (bpm)	≤8		9–11	12–20		21–24	≥25
T (°C)	≤35		35.1–36	36.1–38	38.1–39	≥39.1	
SBP (mmHg)	≤90	91–100	101–110	111–219			≥220
SpO_2_ (%) Scale 1	≤91	92–93	94–95	≥96			
SpO_2_ (%) Scale 2 ^1^	≤83	84–85	86–87	88–92 ≥93 air	93–94 Oxygen	95–96 Oxygen	≥97 Oxygen
Air oxygen		Oxygen		Air			
AVPU (scale)				A			V, P, U
**qSOFA**			**1**				
BR (bpm)			≥22				
SBP (mmHg)			≤100				
GCS (points)			≤13				
**MREMS**	**0**	**1**	**2**	**3**	**4**	**5**	**6**
Pulse (bpm)	70–109		55–69 110–139	40–54 140–179	<39 >179		
BR (bpm)	12–24	10–11 25.34	6–9	35–49	<5 >49		
SBP (mmHg)	70–109		50–69 110–129	130–159	≤49 >159		
SpO_2_ (%)	>89	88–89		75–85	<75		
GCS (scale)	14–15	11–13	8–10	5–7	3–4		
Age (years old)	<45		45–54	55–64		65–74	>74
**RAPS**	**0**	**1**	**2**	**3**	**4**	**5**	**6**
Pulse (bpm)	70–109		55–69 110–139	40–54 140–179	<39 >179		
BR (bpm)	12–24	10–11 25.34	6–9	35–49	<5 >49		
MAP (mmHg)	70–109		50–69 110–129	130–159	≤49 >159		
GCS (scale)	14–15	11–13	8–10	5–7	3–4		

^1^ In patients with hypercapnic respiratory insufficiency, scale 2 should be used to weight the oxygen saturation score. NEWS2: National Early Warning Score 2; qSOFA: quick sequential organ failure assessment score; MREMS: Modified Rapid Emergency Medicine Score; RAPS: Rapid Acute Physiology Score; BR: Breathing rate; T: temperature; SBP: Systolic blood pressure; SpO: Oxygen saturation; AVPU: alert, verbal, pain, unresponsive; GCS: Glasgow coma scale; MAP: Mean arterial pressure.

**Table 2:** Baseline patients’ characteristics based on severe acute respiratory syndrome coronavirus 2 (SARS-CoV-2) infection.

	Total Cohort (*n* = 663)	SARS-CoV-2 (*n* = 261)	Non-SARS-CoV-2 (*n* = 402)	*p* Value
Outcomes, mortality				
2-days	55 (8.3)	32 (12.3)	23 (5.7)	0.004
Demographic characteristics				
Sex, female	341 (51.4)	141 (54.0)	200 (49.8)	0.282
Age (years)	82 (70–88)	80 (69–88)	83 (70–88)	0.348
Age groups (years)				
18–49	53 (8.0)	23 (8.8)	30 (7.5)	
50–74	160 (24.1)	65 (24.9)	95 (23.6)	0.476
≥ 75	446 (67.3)	171 (65.5)	275 (68.4)	0.611
Ambulance				
BLS	617 (93.1)	248 (95.0)	369 (91.8)	
ALS	46 (6.9)	13 (5.0)	33 (8.2)	0.114
Nursing home	300 (45.2)	119 (45.6)	181 (45.0)	0.886
Clinical characteristics				
BR (bpm)	16 (12–24)	17 (12–25)	16 (12–23)	0.133
Saturation (%)	95 (91–98)	94 (89–97)	96 (92–98)	0.002
Suppl. O_2_	120 (18.1)	41 (15.7)	79 (19.7)	0.198
SBP (mmHg)	127 (112–146)	128 (116–148)	126 (108–145)	0.056
MBP (mmHg)	88 (79–101)	89 (82–102)	87 (77–100)	0.076
Heart rate (bpm)	86 (73–101)	95 (75–100)	86 (73–101)	0.880
T (°C)	36.4 (36.0–37.0)	36.4 (36.0–37.0)	36.4 (36.0–36.9)	0.953
GCS points)	15 (14–15)	15 (14–15)	15 (14–15)	0.781
CACI (points)	5 (4–7)	5 (3–6)	6 (4–8)	0.001
Inpatients	531 (80.1)	215 (82.4)	316 (78.6)	0.236
ICU	28 (4.2)	19 (7.3)	9 (2.2)	0.002

Values expressed as total number (fraction) and medians (25th percentile–75th percentile) as appropriate. SARS-CoV-2: severe acute respiratory syndrome coronavirus 2; BLS: basic life support; ALS: advanced life support; BR: breathing rate; Suppl. O_2_: supplemental oxygen; SBP: systolic blood pressure; MBP: mean blood pressure; T: temperature; GCS: Glasgow coma scale; CACI: Charlson age comorbidity index; ICU: intensive care unit.

**Table 3:** Scoring system based on two-day mortality.

	SARS-CoV-2 (*n* = 261)			Non-SARS-CoV-2 (*n* = 402)		
	Survivors (*n* = 229)	Nonsurvivors (*n* = 32)	*p* Value	Survivors (*n* = 379)	Nonsurvivors (*n* = 23)	*p* Value
Demographic characteristics						
Sex, female	123 (53.7)	18 (56.3)	0.788	187 (49.3)	13 (56.5)	0.505
Age (years)	79 (68–88)	85 (79–92)	0.031	82 (69–88)	86 (80–92)	0.006
Nursing home	98 (42.8)	21 (65.6)	0.015	164 (43.3)	17 (73.9)	0.004
Clinical characteristics						
BR (bpm)	16 (12–25)	24 (12–31)	0.023	16 (12–23)	22 (12–28)	0.090
Saturation (%)	95 (90–97)	89 (84–96)	0.009	96 (92–98)	92 (81–97)	0.017
Suppl. O_2_	29 (12.7)	12 (37.5)	<0.001	65 (17.2)	14 (60.1)	<0.001
SBP (mmHg)	128 (117–146)	129 (112–149)	0.188	128 (111–145)	102 (88–122)	0.003
MBP (mmHg)	90 (82–102)	89 (77–102)	0.451	88 (78–101)	71 (62–92)	0.005
Heart rate (bpm)	85 (75–98)	86 (66–108)	0.848	83 (73–101)	88 (50–108)	0.943
T (°C)	36.4 (36.0–37.0)	36.2 (36.0–37.3)	0.285	36.4 (36.0–36.9)	36.4 (36.0–37.4)	0.400
GCS points)	15 (14–15)	13 (8–15)	0.001	15 (14–15)	10 (10–14)	<0.001
EWS (points)						
NEWS2	4 (2–7)	10 (5–12)	<0.001	4 (2–6)	11 (7–13)	<0.001
qSOFA	1 (0–1)	2 (1–2)	<0.001	1 (0–1)	2 (1–2)	<0.001
MREMS	5 (4–7)	9 (6–12)	<0.001	5 (4–7)	10 (8–13)	<0.001
RAPS	1 (1–3)	4 (1–5)	<0.001	2 (0–3)	5 (4–7)	<0.001
CACI (points)	5 (3–6)	6 (5–8)	0.002	6 (4–8)	7 (6–11)	0.003
Inpatients	184 (80.3)	31 (96.9)	0.022	294 (77.6)	22 (95.7)	0.040
ICU	19 (8.3)	0	0.091	7 (1.8)	2 (8.7)	0.031

Values expressed as total number (fraction) and medians (25th percentile–75th percentile) as appropriate. SARS-CoV-2: severe acute respiratory syndrome coronavirus 2; BLS: basic life support; ALS: advanced life support; BR: breathing rate; Suppl. O_2_: supplemental oxygen; SBP: systolic blood pressure; MBP: mean blood pressure; T: temperature; GCS: Glasgow coma scale; EWS: early warning scores; NEWS2: National Early Warning Score 2; qSOFA: quick sequential organ failure assessment score; MREMS: Modified Rapid Emergency Medicine Score; RAPS: Rapid Acute Physiology Score; CACI: Charlson age comorbidity index; ICU: intensive care unit.

**Table 4:** Area under the receiver operating characteristics for all outcomes analyzed by two-day mortality.

	NEWS2	qSOFA	MREMS	RAPS
Global	0.825 (0.75–0.89)	0.761 (0.68–0.83)	0.803 (0.73–0.87)	0.775 (0.70–0.85)
SARS-CoV-2	0.804 (0.71–0.89)	0.736 (0.63–0.83)	0.764 (0.66–0.86)	0.750 (0.64–0.82)
Non-SARS-CoV-2	0.863 (0.76–0.95)	0.799 (0.68–0.91)	0.860 (0.76–0.95)	0.815 (0.70–0.92)

Bracketed numbers indicate 95% confidence intervals. In all cases, *p* values < 0.001. NEWS2: National Early Warning Score 2; qSOFA: quick sequential organ failure assessment score; MREMS: Modified Rapid Emergency Medicine Score; RAPS: Rapid Acute Physiology Score; SARS-CoV-2: severe acute respiratory syndrome coronavirus 2.

**Table 5:** Combined sensitivity and specificity (Youden’s test) for all scores analyzed.

	Cutoff	Se	Sp	PPV	NPV	LR (+)	LR (−)	OR
NEWS2								
Global	7	78.2 (65.6–87.1)	73.4 (69.7–76.7)	21.0 (16.0–27.1)	97.4 (95.5–98.5)	2.93 (2.42–3.56)	0.30 (0.18–0.49)	9.87 (5.08–19.1)
SARS-CoV-2	8	71.9 (54.6–84.4)	76.4 (70.5–81.5)	29.9 (20.8–40.8)	95.1 (91.0–97.4)	3.05 (2.22–4.19	0.37 (0.21–0.65)	8.28 (3.62–18.9)
Non-SARS-CoV-2	7	87.0 (67.9–95.5)	75.2 (70.6–79.3)	17.5 (11.7–25.6)	99.0 (97.0–99.6)	3.51 (2.77–4.44)	0.17 (0.06–0.50)	20.2 (5.87–69.5)
qSOFA								
Global	2	58.2 (45.0–70.3)	81.4 (78.1–84.3)	22.1 (16.1–29.5)	95.6 (93.4–97.0)	3.13 (2.37–4.14)	0.51 (0.37–0.71)	6.09 (3.43–10.8)
SARS-CoV-2	2	53.1 (36.4–69.1)	82.1 (76.6–86.5)	29.3 (19.2–42.0)	92.6 (88.2–95.5)	2.97 (1.93–4.55)	0.57 (0.39–0.84)	5.20 (2.40–11.2)
Non-SARS-CoV-2	2	65.2 (44.9–81.2)	81.0 (76.7–84.6)	17.2 (10.7–26.5)	97.5 (95.1–98.7)	3.43 (2.39–4.94)	0.43 (0.24–0.76)	7.99 (3.26–19.5)
MREMS								
Global	8	69.1 (56.0–79.7)	80.6 (77.3–83.5)	24.4 (18.3–31.7)	96.6 (94.7–97.9)	3.56 (2.80–4.52)	0.38 (0.26–0.57)	9.28 (5.06–17.1)
SARS-CoV-2	9	59.4 (42.3–74.5)	87.8 (82.9–91.4)	40.4 (27.6–54.7)	93.9 (89.9–96.4)	4.86 (3.10–7.72)	0.46 (0.30–0.71)	10.4 (4.67–23.5)
Non-SARS-CoV-2	8	78.3 (58.1–90.3)	81.8 (77.6–85.4)	20.7 (13.5–30.4)	98.4 (96.3–99.3)	4.30 (3.17–5.82)	0.27 (0.12–0.58)	16.1 (5.81–45.1)
RAPS								
Global	4	67.3 (54.1–78.2)	84.2 (81.1–86.9)	27.8 (20.9–36.0)	96.6 (94.7–97.8)	4.26 (3.28–5.53)	0.39 (0.26–0.57)	10.9 (5.99–20.1)
SARS-CoV-2	4	59.4 (42.3–74.5)	84.3 (79.0–88.4)	34.5 (23.4–47.7)	93.7 (89.5–96.3)	3.78 (2.49–5.72)	0.48 (0.31–0.74)	7.84 (3.56–17.2)
Non-SARS-CoV-2	4	78.3 (58.1–90.3)	84.2 (80.2–87.5)	23.1 (15.1–33.6)	98.5 (96.4–99.3)	4.94 (3.60–6.79)	0.26 (0.12–0.56)	19.1 (6.84–53.5)

Bracketed numbers indicate 95% confidence intervals. NEWS2: National Early Warning Score 2; qSOFA: quick sequential organ failure assessment score; MREMS: Modified Rapid Emergency Medicine Score; RAPS: Rapid Acute Physiology Score; SARS-CoV-2: severe acute respiratory syndrome coronavirus 2; Se: Sensitivity; Sp: Specificity; PPV: Positive predictive value: NPV: Negative predictive value; LR: Likelihood ratio; OR: Odds ratio.

**Table 6:** Comparison between curves for all outcomes analyzed by a Delong test.

		NEWS	qSOFA	RAPS	MREMS
Global	NEWS				
	qSOFA	**0.0006**			
	RAPS	**0.049**	0.651		
	MREMS	0.272	0.150	0.243	
SARS-CoV-2	NEWS				
	qSOFA	**0.019**			
	RAPS	0.075	0.739		
	MREMS	0.171	0.510	0.697	
Non-SARS-CoV-2	NEWS				
	qSOFA	**0.002**			
	RAPS	0.299	0.757		
	MREMS	0.924	0.096	0.081	

Statistically significant results are highlighted in bold. NEWS2: National Early Warning Score 2; qSOFA: quick sequential organ failure assessment score; MREMS: Modified Rapid Emergency Medicine Score; RAPS: Rapid Acute Physiology Score; SARS-CoV-2: severe acute respiratory syndrome coronavirus. Bold numbers mean statistical significance.

## Data Availability

The data presented in this study are available on request from the corresponding author.

## References

[1] Maves R.C., Downar J., Dichter J.R., Hick J.L., Devereaux A., Geiling J.A., Kissoon N., Rubinson L.L., Hanfling D., Hodge J.G. (2020). Triage of Scarce Critical Care Resources in COVID-19 an Implementation Guide for Regional Allocation: An Expert Panel Report of the Task Force for Mass Critical Care and the American College of Chest Physicians. Chest.

[2] Jaffe E., Sonkin R., Strugo R., Zerath E. (2020). Evolution of emergency medical calls during a pandemic—An emergency medical service during the COVID-19 outbreak. Am. J. Emerg. Med..

[3] Zhou F., Yu T., Du R., Fan G., Liu Y., Liu Z., Xiang J., Wang Y., Song B., Gu X. (2020). Clinical course and risk factors for mortality of adult inpatients with COVID-19 in Wuhan, China: A retrospective cohort study. Lancet.

[4] Tien H., Sawadsky B., Lewell M., Peddle M., Durham W. (2020). Critical care transport in the time of COVID-19. CJEM.

[5] Mileder L.P., Schüttengruber G., Prattes J., Wegscheider T. (2020). Simulation-based training and assessment of mobile pre-hospital SARS-CoV-2 diagnostic teams in Styria, Austria. Medicine.

[6] Phua J., Weng L., Ling L., Egi M., Lim C.M., Divatia J.V., Shrestha B.R., Arabi Y.M., Med N.M., Gomersall C.D. (2020). Intensive care management of coronavirus disease 2019 (COVID-19): Challenges and recommendations. Lancet Respir. Med..

[7] Seymour C.W., Liu V.X., Iwashyna T.J., Brunkhorst F.M., Rea T.D., Scherag A., Rubenfeld G., Kahn J.M., Shankar-Hari M., Singer M. (2016). Assessment of Clinical Criteria for Sepsis: For the Third International Consensus Definitions for Sepsis and Septic Shock (Sepsis-3). JAMA.

[8] Hendren N.S., Drazner M.H., Bozkurt B., Cooper L.T. (2020). Description and Proposed Management of the Acute COVID-19 Cardiovascular Syndrome. Circulation.

[9] Liu F.Y., Sun X.L., Zhang Y., Ge L., Wang J., Liang X., Li J.-F., Wang C.-L., Xing Z.-T., Chhetri J.K. (2020). Evaluation of the Risk Prediction Tools for Patients with Coronavirus Disease 2019 in Wuhan, China: A Single-Centered, Retrospective, Observational Study. Crit. Care Med..

[10] Panday R.S.N., Minderhoud T.C., Alam N., Nanayakkara P.W.B. (2017). Prognostic value of early warning scores in the emergency department (ED) and acute medical unit (AMU): A narrative review. Eur. J. Intern. Med..

[11] Martín-Rodríguez F., López-Izquierdo R., Del Vegas C.P., Benito J.F.D., Rodríguez V.C., Rasilla M.N.D., Conty J.L.M., Scar A.M., de la Torre S.O., Martín V.M. (2019). Accuracy of National Early Warning Score 2 (NEWS2) in Prehospital Triage on In-Hospital Early Mortality: A Multi-Center Observational Prospective Cohort Study. Prehosp. Disaster Med..

[12] Spencer W., Smith J., Date P., de Tonnerre E., Taylor D.M. (2019). Determination of the best early warning scores to predict clinical outcomes of patients in the emergency department. Emerg. Med. J..

[13] Spångfors M., Molt M., Samuelson K. (2020). National Early Warning Score: A survey of registered nurses’ perceptions, experiences and barriers. J. Clin. Nurs..

[14] Gidari A., De Socio G.V., Sabbatini S., Francisci D. (2020). Predictive value of National Early Warning Score 2 (NEWS2) for intensive care unit admission in patients with SARS-CoV-2 infection. Infect. Dis. (Lond.).

[15] Khwannimit B., Bhurayanontachai R., Vattanavanit V. (2019). Comparison of the accuracy of three early warning scores with SOFA score for predicting mortality in adult sepsis and septic shock patients admitted to intensive care unit. Heart Lung.

[16] Sridhar S., Schmid A., Biziyaremye F., Hodge S., Patient N., Wilson K. (2020). Implementation of a Pediatric Early Warning Score to Improve Communication and Nursing Empowerment in a Rural District Hospital in Rwanda. Glob. Health Sci. Pract..

[17] Russell S., Stocker R., Barker R.O., Liddle J., Adamson J., Hanratty B. (2020). Implementation of the National Early Warning Score in UK care homes: A qualitative evaluation. Br. J. Gen. Pract..

[18] Foy K.E., Pearson J., Kettley L., Lal N., Blackwood H., Bould M.D. (2020). Four early warning scores predict mortality in emergency surgical patients at University Teaching Hospital, Lusaka: A prospective observational study. Can. J. Anaesth..

[19] Haegdorens F., Monsieurs K.G., De Meester K., Van Bogaert P. (2020). The optimal threshold for prompt clinical review: An external validation study of the national early warning score. J. Clin. Nurs..

[20] Ehara J., Hiraoka E., Hsu H.C., Yamada T., Homma Y., Fujitani S. (2019). The effectiveness of a national early warning score as a triage tool for activating a rapid response system in an outpatient setting: A retrospective cohort study. Medicine.

[21] Parshuram C.S., Dryden-Palmer K., Farrell C., Gottesman R., Gray M., Hutchison J.S., Helfaer M., Hunt E.A., Joffe A.R., Lacroix J. (2018). Effect of a Pediatric Early Warning System on All-Cause Mortality in Hospitalized Pediatric Patients: The EPOCH Randomized Clinical Trial. JAMA.

[22] Paternina-Caicedo A., Miranda J., Bourjeily G., Levinson A., Dueñas C., Bello-Muñoz C., Rojas-Suarez J.A. (2017). Performance of the Obstetric Early Warning Score in critically ill patients for the prediction of maternal death. Am. J. Obstet. Gynecol..

[23] Covino M., Sandroni C., Santoro M., Sabia L., Simeoni B., Bocci M.G., Ojetti V., Candelli M., Antonelli M., Gasbarrini A. (2020). Predicting intensive care unit admission and death for COVID-19 patients in the emergency department using early warning scores. Resuscitation.

[24] De Nardo P., Gentilotti E., Mazzaferri F., Cremonini E., Hansen P., Goossens H., Tacconelli E. (2020). Multi-Criteria Decision Analysis to prioritize hospital admission of patients affected by COVID-19 in low-resource settings with hospital-bed shortage. Int. J. Infect. Dis..

[25] Yang P., Wang P., Song Y., Zhang A., Yuan G., Cui Y. (2020). A retrospective study on the epidemiological characteristics and establishment of an early warning system of severe COVID-19 patients. J. Med. Virol..

[26] Pimentel M.A.F., Redfern O.C., Hatch R., Young J.D., Tarassenko L., Watkinson P.J. (2020). Trajectories of vital signs in patients with COVID-19. Resuscitation.

[27] Martín-Rodríguez F., Castro-Villamor M.Á., Del Vegas C.P., Martín-Conty J.L., Mayo-Iscar A., Benito J.F.D., del Brio I.P., Arnillas-Gómez P., Escudero-Cuadrillero C., López-Izquierdo R. (2019). Analysis of the early warning score to detect critical or high-risk patients in the prehospital setting. Intern. Emerg. Med..

[28] Shankar-Hari M., Phillips G.S., Levy M.L., Seymour C.W., Liu V.X., Deutschman C.S., Angus D.C., Rubenfeld G.D., Singer M. (2016). Developing a New Definition and Assessing New Clinical Criteria for Septic Shock: For the Third International Consensus Definitions for Sepsis and Septic Shock (Sepsis-3). JAMA.

[29] Singer M., Deutschman C.S., Seymour C.W., Shankar-Hari M., Annane D., Bauer M., Bellomo R., Bernard G.R., Chiche I.-D., Coopersmith C.M. (2016). The Third International Consensus Definitions for Sepsis and Septic Shock (Sepsis-3). JAMA.

[30] Kivipuro M., Tirkkonen J., Kontula T., Solin J., Kalliomäki J., Pauniaho S.L., Huhtala H., Yli-Hankala A., Hoppu S. (2018). National early warning score (NEWS) in a Finnish multidisciplinary emergency department and direct vs. late admission to intensive care. Resuscitation.

[31] Miller R.T., Nazir N., McDonald T., Cannon C.M. (2017). The modified rapid emergency medicine score: A novel trauma triage tool to predict in-hospital mortality. Injury.

[32] Chang S.H., Hsieh C.H., Weng Y.M., Hsieh M.S., Goh Z.N.L., Chen H.Y., Chang T., Ng C.-J., Seak J.C.-Y., Seak C.-K. (2018). Performance Assessment of the Mortality in Emergency Department Sepsis Score, Modified Early Warning Score, Rapid Emergency Medicine Score, and Rapid Acute Physiology Score in Predicting Survival Outcomes of Adult Renal Abscess Patients in the Emergency D. BioMed Res. Int..

[33] McLymont N., Glover G.W. (2016). Scoring systems for the characterization of sepsis and associated outcomes. Ann. Transl. Med..

[34] Scott L.J., Redmond N.M., Garrett J., Whiting P., Northstone K., Pullyblank A. (2019). Distributions of the National Early Warning Score (NEWS) across a healthcare system following a large-scale roll-out. Emerg. Med. J..

[35] Campbell V., Conway R., Carey K., Tran K., Visser A., Gifford S., McLanders M., Edelson D., Churpek M. (2020). Predicting clinical deterioration with Q-ADDS compared to NEWS, Between the Flags, and eCART track and trigger tools. Resuscitation.

[36] Myrstad M., Ihle-Hansen H., Tveita A.A., Andersen E.L., Nygård S., Tveit A., Berge T. (2020). National Early Warning Score 2 (NEWS2) on admission predicts severe disease and in-hospital mortality from Covid-19—A prospective cohort study. Scand. J. Trauma Resusc. Emerg. Med..

[37] Jang J.G., Hur J., Hong K.S., Lee W., Ahn J.H. (2020). Prognostic Accuracy of the SIRS, qSOFA, and NEWS for Early Detection of Clinical Deterioration in SARS-CoV-2 Infected Patients. J. Korean Med. Sci..

[38] Sixt T., Moretto F., Devilliers H., Abdallahoui M., Eberl I., Rogier T., Duong M., Salmon-Rousseau A., Mahy S., Buisson M. (2020). The usefulness of NEWS2 at day 7 of hospitalization in predicting COVID-19 evolution and as an early endpoint in therapeutic trials. J. Infect..

[39] Hu H., Yao N., Qiu Y. (2020). Comparing Rapid Scoring Systems in Mortality Prediction of Critically Ill Patients with Novel Coronavirus Disease. Acad. Emerg. Med..

[40] Hu H., Yao N., Qiu Y. (2020). Predictive value of five early warning scores for critical novel coronavirus disease. Disaster Med. Public Health Prep..

[41] Weng Z., Chen Q., Li S., Li H., Zhang Q., Lu S., Wu L., Xiong L., Mi B., Liu D. (2020). ANDC: An early warning score to predict mortality risk for patients with Coronavirus Disease 2019. J. Transl. Med..

[42] Li L.Q., Huang T., Wang Y.Q., Wang Z.P., Liang Y., Huang T.B., Zhang H.-Y., Sun W., Wang Y. (2020). COVID-19 patients’ clinical characteristics, discharge rate, and fatality rate of meta-analysis. J. Med. Virol..

[43] Lapostolle F., Schneider E., Vianu I., Dollet G., Roche B., Berdah J., Michel J., Goix L., Chanzy E., Petrovic T. (2020). Clinical features of 1487 COVID-19 patients with outpatient management in the Greater Paris: The COVID-call study. Intern. Emerg. Med..

[44] Churpek M.M., Snyder A., Han X., Sokol S., Pettit N., Howell M.D., Edelson D.P. (2017). Quick Sepsis-related Organ Failure Assessment, Systemic Inflammatory Response Syndrome, and Early Warning Scores for Detecting Clinical Deterioration in Infected Patients outside the Intensive Care Unit. Am. J. Respir. Crit. Care Med..

[45] Keep J.W., Messmer A.S., Sladden R., Burrell N., Pinate R., Tunnicliff M., Glucksman E. (2016). National early warning score at Emergency Department triage may allow earlier identification of patients with severe sepsis and septic shock: A retrospective observational study. Emerg. Med. J..

[46] Haegdorens F., Monsieurs K.G., De Meester K., Van Bogaert P. (2019). An intervention including the national early warning score improves patient monitoring practice and reduces mortality: A cluster randomized controlled trial. J. Adv. Nurs..

[47] Assaf D., Gutman Y., Neuman Y., Segal G., Amit S., Gefen-Halevi S., Shilo N., Epstein A., Mor-Cohen R., Biber A. (2020). Utilization of machine-learning models to accurately predict the risk for critical COVID-19. Intern. Emerg. Med..

[48] Knight S.R., Ho A., Pius R., Buchan I., Carson G., Drake T.M., Dunning J., Fairfield C.J., Green C.A., Halpin S. (2020). Risk stratification of patients admitted to hospital with covid-19 using the ISARIC WHO Clinical Characterisation Protocol: Development and validation of the 4C Mortality Score. BMJ.

[49] Hu C., Liu Z., Jiang Y., Shi O., Zhang X., Xu K., Suo C., Wang Q., Song Y., Yu K. (2020). Early prediction of mortality risk among patients with severe COVID-19, using machine learning. Int. J. Epidemiol..

[50] Haimovich A.D., Ravindra N.G., Stoytchev S., Young H.P., Wilson F.P., van Dijk D., Schulz W.L., Taylor R. (2020). AMichel. Development and Validation of the Quick COVID-19 Severity Index: A Prognostic Tool for Early Clinical Decompensation. Ann. Emerg. Med..

[51] Rodriguez-Nava G., Yanez-Bello M.A., Trelles-Garcia D.P., Chung C.W., Friedman H.J., Hines D.W. (2021). Performance of the quick COVID-19 severity index and the Brescia-COVID respiratory severity scale in hospitalized patients with COVID-19 in a community hospital setting. Int. J. Infect. Dis..

